# Rapid Translation of the Early Coding Region Promotes Premature Transcription Termination, Reducing Protein Expression in *Escherichia coli*

**DOI:** 10.1111/mmi.70113

**Published:** 2026-09-11

**Authors:** Steen Pedersen, Alberte Honoré Jepsen, Bertil Gummesson, Namiko Mitarai, Kim Sneppen, Sine Lo Svenningsen

**Affiliations:** ^1^ Department of Biology University of Copenhagen Copenhagen N Denmark; ^2^ Niels Bohr Institute University of Copenhagen Copenhagen N Denmark

## Abstract

Slowly translated codons are overrepresented among the first approximately 30 codons of natural genes across all domains of life, yet the functional basis for this conserved feature remains incompletely understood. Using the *Escherichia coli lacZ* gene as a model, we previously showed that insertion of fast‐translated codons into the early coding region dramatically reduces β‐galactosidase production by increasing premature transcription termination and decreasing mRNA stability. Here we show that premature transcription termination events occur several nucleotides downstream of the fast‐codon inserts, at positions coinciding with previously characterized Rho‐dependent intragenic termination sites in *lacZ*. To exclude the possibility that the specific amino acid sequence of the inserts—rather than their translation speed—was responsible for termination, we constructed two additional *lacZ* variants with distinct fast‐translated sequences, including one replacing the 30 earliest *lacZ* codons with their fastest synonymous alternatives. Both variants showed premature transcription termination of comparable magnitude to the original inserts, demonstrating that it is the high translation rate itself which causes premature termination. Analysis of a conservative set of seven high‐confidence fast‐translated codons across natural highly expressed genes and the synthetic constructs revealed that a run of consecutive fast codons is the feature that most clearly distinguishes the terminating sequences from natural genes. We propose that excessively rapid synthesis of the N‐terminal part of a polypeptide may impair its proper entry into the ribosomal exit tunnel, thereby disrupting transcription‐translation coupling and exposing downstream mRNA to Rho‐dependent termination.

## Plain Language Summary

Almost all genes begin with codons that are translated relatively slowly, and the reason why is not fully understood. We replaced the first stretch of codons of a bacterial gene with fast codons and found that transcription then often stopped partway through the gene, so very little protein was made. The slow start may be needed for efficient protein synthesis.

## Graphical abstract

A run of consecutive fast‐translated codons (right) within the first 30 codons of *lacZ* (top) causes loss of the downstream transcript at positions coinciding with previously mapped intragenic Rho‐dependent termination sites (middle), and a corresponding loss of β‐galactosidase activity (bottom). Wildtype *lacZ*, which carries no such run, is unaffected (left).

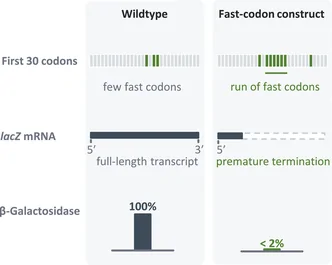

## 1 Introduction

The genetic code is degenerate, with most amino acids encoded by two or more synonymous codons. These codons are not used equally: highly expressed genes show a strong bias toward a specific subset of synonymous codons (Post et al. 1979). To quantify this bias, Sharp and Li (1987) developed the codon adaptation index (CAI), which ranges from unbiased codon usage (low CAI) to a strong preference for the codons favored in highly expressed genes (high CAI). Genes using preferentially non‐biased codons are translated at about half the rate of highly expressed genes in *Escherichia coli* (Pedersen 1984).

Strikingly, the first approximately 30 codons of natural genes in *E. coli* have a low CAI irrespective of the expression level of the gene (Bulmer 1988), a feature which is evolutionarily conserved across bacteria, archaea, and eukaryotes (Tuller et al. 2010). It is therefore very likely that the first segment of most mRNAs is translated at a lower rate than the remaining part of the mRNA.

To discriminate whether these early slowly translated codons were selected for or if the later fast‐translated codons were selected for and the early codons were drifting toward nonbiased codons as a result of no selection, the frequency of shift to synonymous codons was investigated in *E. coli and Salmonella typhimurium*. The results suggested a significant direct selection for slowly translated early codons (Eyre‐Walker and Bulmer 1993; Mitarai et al. 2008). One may therefore assume that there is a benefit to relatively slow translation of early codons. We (Mitarai et al. 2008) and others (Tuller et al. 2010) suggested that one function of the preferential use of slowly translated codons in the early part of genes was to minimize ribosome queue formation on the later part of the mRNA.

Here, we suggest an additional function for slow early translation of mRNAs, demonstrated by the *E. coli* lac operon. We previously used modeling to predict that the distribution of fast and slow codons in the early part of the mRNA had a large impact on mRNA half‐life (Mitarai et al. 2008; Pedersen et al. 2011). To verify this experimentally, we inserted fast translated codons in the early part of the *lacZ* gene of *E. coli* and observed up to 100‐fold reduction in β‐galactosidase expression. The reduced protein levels were the result of an up to 10‐fold reduction of the mRNA half‐life and an estimated increase in premature transcription termination events by up to 90% (Pedersen et al. 2019). Transcription and translation are coupled in *E. coli* , where the RNA polymerase is physically tethered to the leading ribosome via a NusG bridge that contacts ribosomal protein S10 and the β‐subunit of RNA polymerase (Burmann et al. 2010; Proshkin et al. 2010). If this contact is broken, for instance because the leading ribosome terminates translation at a stop codon and releases the mRNA while RNA polymerase continues transcription, the nascent mRNA becomes accessible to termination factor Rho, which causes transcription termination (Roberts 1969; Molodtsov et al. 2023; Murayama et al. 2023). In the case of the lac operon, a polar stop codon in *lacZ* reduced synthesis of LacY and LacA proteins (Newton et al. 1965). Premature transcription termination was an unexpected consequence of the fast‐translated early codons in our constructs because most transcription of the wildtype *lacZYA* genes continues to the end of the operon (Murakawa et al. 1991), and because the inserted sequences bear no resemblance to a stem‐loop poly‐U transcription termination sequence.

We now show that premature transcription termination is caused by the early fast translated codons but takes place in the wildtype part of the *lacZ* sequence, substantially downstream of the inserted sequences. The main termination sites coincide with Rho‐dependent intragenic termination sites previously shown to terminate in vivo when the nascent mRNA is not co‐translated (Ruteshouser and Richardson 1989). As a working model, we hypothesize that fast translation of the early region of *lacZ* mRNA may cause premature transcription termination because the rapidly synthesized nascent polypeptide does not properly enter the peptide exit tunnel and thereby stalls the ribosome.

## 2 Results

In our previous work (Pedersen et al. 2019), we demonstrated that inserts of fast‐translated codons in the early part of the *lacZ* open reading frame caused a reduction in the amount of enzyme produced from the last gene in the operon, LacA, relative to wildtype. This polar effect was interpreted as premature transcription termination but did not reveal if the termination sites coincided with the fast codon insertions. To first examine if premature transcription termination occurs within the *lacZ* gene, we used RT‐qPCR to determine the levels of the middle and distal regions of *lacZ* mRNA relative to the levels of the 5′ UTR mRNA region upstream of the fast codon inserts. Total RNA was harvested from three *E. coli* strains carrying plasmids with insertions of 7, 14, and 21 fast‐translated codons, respectively, inserted between codon 11 and codon 19 of *lacZ* ^*+*^ (Pedersen et al. 2019), see Figure 1a and Table 2 (Section 4).

**FIGURE 1:**
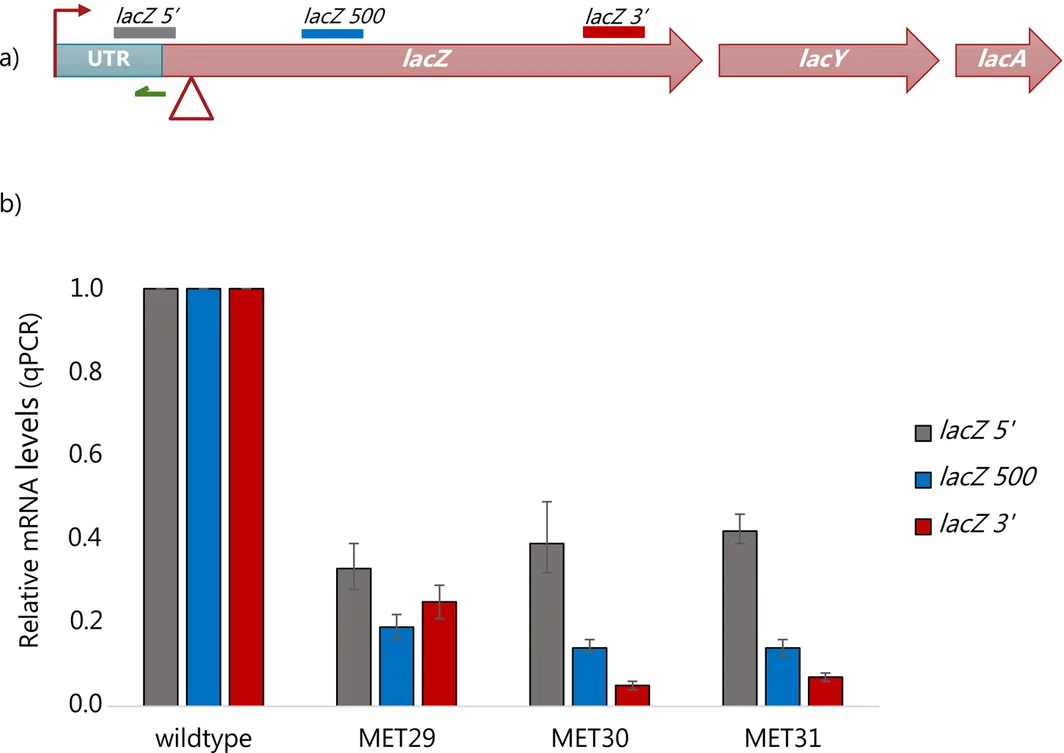
Premature transcription termination in engineered *lacZ* variants containing fast‐translated early codons. (a) LacZ constructs in the MET20 plasmid and its derivatives (Pedersen et al. 2019). Open triangle indicates the insertion of 7 (MET29), 14 (MET30), or 21 (MET31) fast‐translated codons between codon eleven and codon nineteen of *lacZ*. An early *lacZ* qPCR primer pair was used before the insert (*lacZ 5*′, gray), a pair around base 500 (*lacZ 500*, blue), and a pair close to the 3′ end (*lacZ 3*′, red). Green half arrow indicates the binding site for the northern blot probe. The schematic is not drawn to scale. (b) Relative levels of mRNA from the *lacZYA* operon. RNA was harvested after 15 min of IPTG induction from cultures of the indicated *E. coli* strains grown in MOPS minimal medium with 0.2% glycerol as carbon source (see Section 4). The *E. coli* strains carried plasmids with the *lacZYA* variants described for panel (a). Expression levels are presented relative to the wildtype strain (MET20). Origin of the qPCR products *lacZ 5*′ (gray), *lacZ 500* (blue), and *lacZ 3*′ (red) is shown in panel (a). Error bars indicate one standard deviation.

As shown in Figure 1b, levels of the distal part of the *lacZ* mRNA (*lacZ 3*′, red bars) were up to 20‐fold reduced in the strains with inserted fast translated codons relative to wildtype, while the levels of the 5′ UTR region of the mRNA, upstream of the inserted sequences, were reduced by only 2‐ to 2.5‐fold (*lacZ 5*′, gray bars). We expect that the modest reduction in the *lacZ* 5′UTR levels, which is upstream of any insert‐related transcription termination points, is due to a reduced half‐life of the mRNAs with inserts, as demonstrated previously (Pedersen et al. 2019). The stark decreases in levels of the middle and distal qPCR products point to a clear increase in premature transcription termination within the *lacZ* gene in the strains with fast‐translated inserts. For the strains with inserts of 14 or 21 fast‐translated codons where termination is most pronounced, approximately two thirds of the termination events appear to take place within the first ~500 bp of lacZ (between the *lacZ 5*′ and the *lacZ 500* qPCR products) and another third of the termination events take place between the *lacZ 500* and the distal *lacZ 3*′ qPCR products (Figure 1b). Quantitative PCR with primers for *lacA* confirmed that *lacA* mRNA levels were also reduced in the insert mutants (Figure S1), consistent with premature termination. For quantification of termination, we relied on the intragenic *lacZ* qPCR products (Figure 1b) rather than on *lacYA* mRNA levels (see Supporting Information S1).

The inserted sequences are rich in GAA codons encoding glutamic acid. One could argue that this stretch of mainly acidic amino acids, or another property of the specific amino acid or mRNA sequence, may cause transcription termination. We compared the amino acid sequence encoded by the inserts to that of abundant naturally acidic proteins. As shown in Figure 2a, the amino acid composition of the first 30 codons of the strains with fast‐translated inserts is progressively enriched for amino acids with acidic properties relative to naturally occurring N‐terminal peptides. They do not differ significantly in their content of amino acids with basic, hydrophobic, or hydrophilic properties (Figure 2b–d). The construct with the shortest insert of fast‐translated codons, pMET29, shows strong premature termination (about 82% of transcripts; Pedersen et al. 2019), but has a similar number of acidic amino acid sidechains to several highly expressed proteins (e.g., elongation factor TS (*tsf*), ribosomal protein L12 (*rplL*), transcription factor NusA, and the metabolic enzyme enolase (*eno*)), neither of which are known to show premature termination. However, the N‐terminal regions of the proteins encoded by pMET30 and pMET31 are enriched in acidic amino acids compared to those regions of natural acidic proteins. Therefore, we could not confidently conclude that the high degree of premature transcription termination did not relate to the acidic nature of the constructs or a combination of properties we had not analyzed.

**FIGURE 2:**
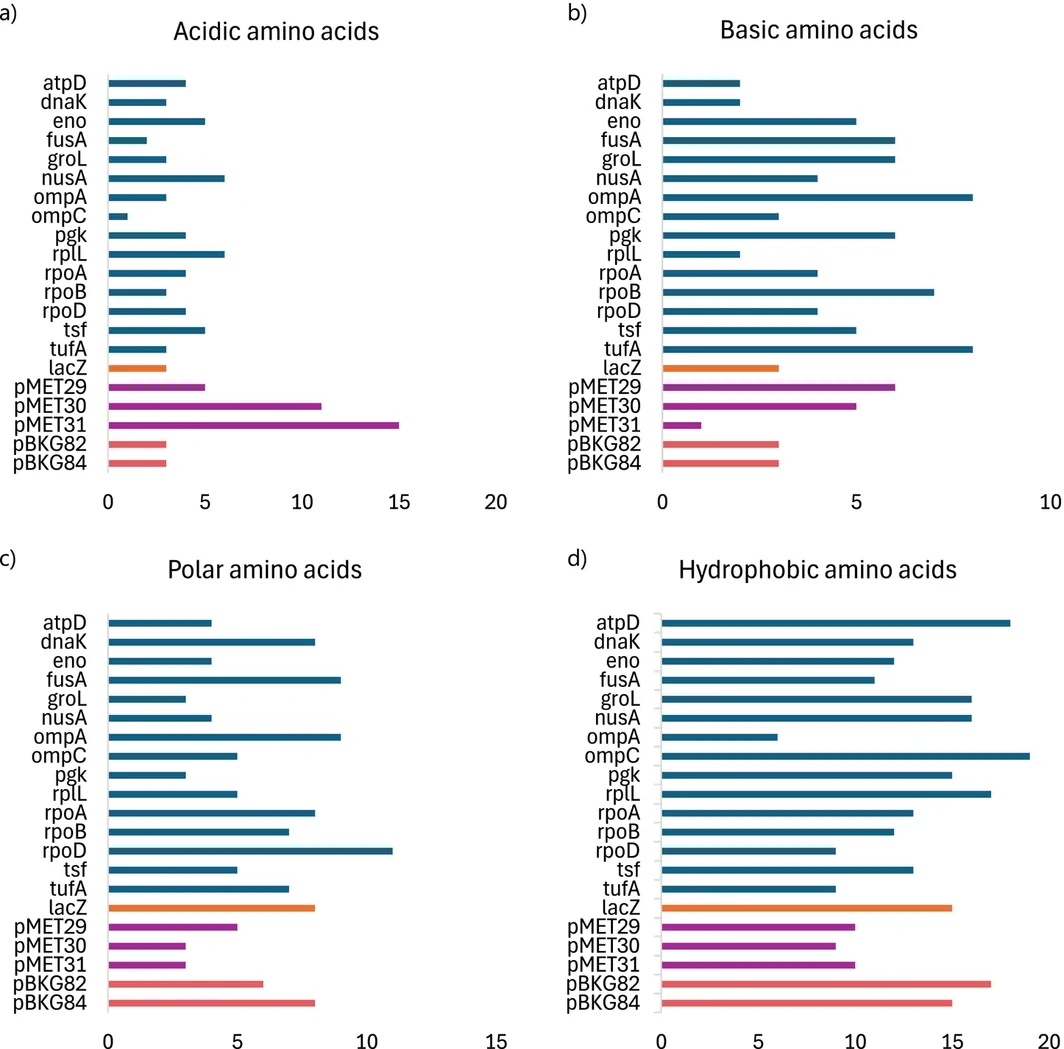
Properties of the initial 30 amino acids of selected natural genes and the constructs used in this work. Number of amino acids with the indicated sidechain properties. We note that Gly was not assigned to any category due to its unique structural properties. (a) Acidic (Asp, Glu). (b) Basic (Lys, Arg, His). (c) Polar (Ser, Thr, Cys, Tyr, Asn, Gln). (d) Hydrophobic (Ala, Val, Ile, Leu, Met, Phe, Trp, Pro).

To test if non‐acidic early fast‐translated sequences in *lacZ* also cause premature transcription termination, we constructed two additional plasmids that use other fast‐translated codons than the MET29‐MET31 series (Figure 3a). First, we replaced *lacZ* codons 5–13 in the plasmid pMAS2 (Sørensen et al. 1989) with nine fast‐translated codons, including seven GCU codons encoding the hydrophobic amino acid alanine. This yielded plasmid pBKG82. Second, we modified the *lacZ* gene in pMAS2, so the first 30 codons are the highest‐CAI synonymous variant of the natural *lacZ* gene, thus expecting the highest achievable translation rate throughout the early region without changing amino acid identities. This yielded plasmid pBKG84. The DNA sequences of the constructs are listed in Table 2 (see Section 4).

**FIGURE 3:**
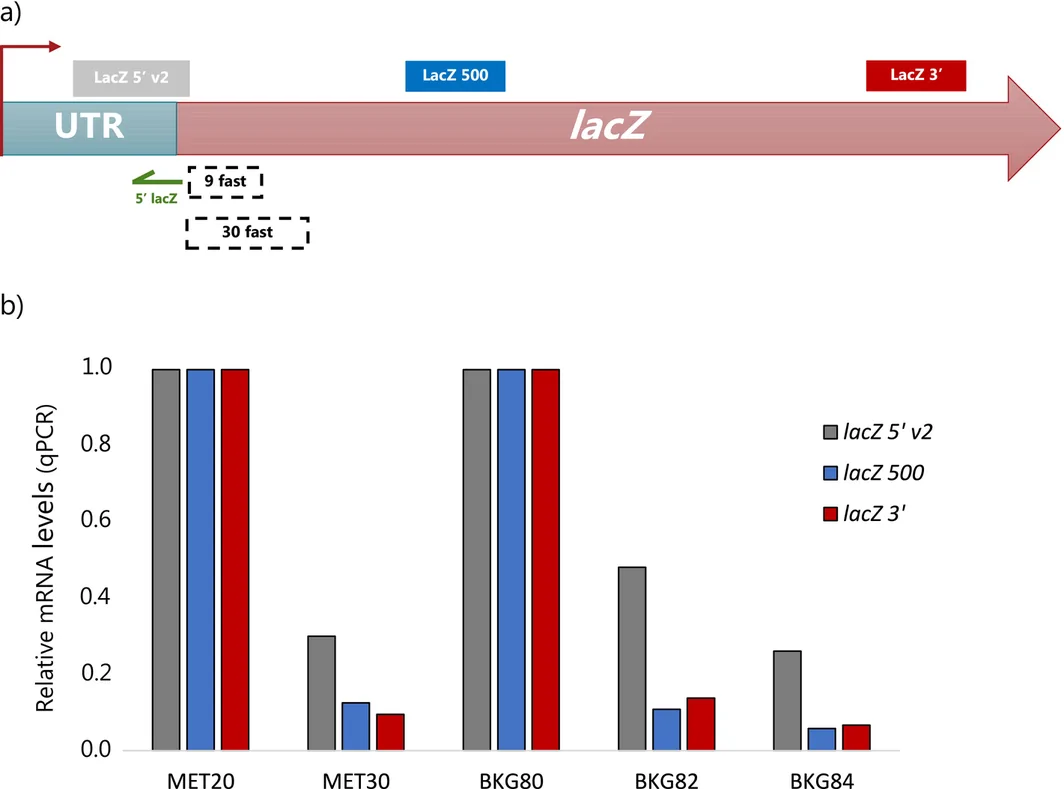
Premature transcription termination in engineered *lacZ* variants containing fast‐translated early codons. (a) *lacZ* constructs in pBKG80 plasmids and its derivatives. Dashed rectangles indicate the sequence changes to fast‐translated variants from codon five to thirteen (9 fast, BKG82) and 30 synonymous fast‐translated codons from codon one to thirty (30 fast, BKG84). An early *lacZ* qPCR primer pair was used before the inserts (*lacZ 5*′*v2*, gray), a pair around base 500 (*lacZ 500*, blue) and a pair close to the 3′ end (*lacZ 3*′, red). A green half arrow indicates the binding site for the northern blot probe. Note the *5*′ *lacZ* primer set used here (*lacZ 5*′ *v2*) is different from the primer set used to quantify the 5′ region of *lacZ* in Figure 1 (*lacZ 5*′) to ensure primer complementarity with the new constructs. The schematic is not drawn to scale. (b) Relative *lacZ* mRNA levels measured by RT‐qPCR on RNA harvested from the indicated cultures 40 min after induction by IPTG. Bacterial cultures were grown in MOPS minimal medium with 0.2% proline as the carbon source (see Section 4). Levels of *lacZ* mRNA were normalized to the mRNA from housekeeping gene *cysG* and are presented relative to the corresponding wildtype reference strain carrying the wildtype version of *lacZ* (MET20 and BKG80, respectively). Locations of the qPCR products *lacZ 5*′ *v2* (gray), *lacZ 500* (blue), and *lacZ 3*′ (red) are shown in panel (a).

Table 1 shows the fully IPTG‐induced β‐galactosidase activity for the two strains carrying wildtype variants of *lacZ* (MET20 and BKG80), and three variants with fast early‐translated codons: MET30, BKG82, and BKG84. Beta‐galactosidase activities of the uninduced strains were very low (< 10 Miller units), and enzyme activity was induced by IPTG in all the strains. However, the induced enzyme activity was severely reduced in all strains containing fast‐translated variants of *lacZ*, suggesting poor protein production.

**TABLE 1:** β‐Galactosidase activity of *E. coli* strains carrying early fast‐translated codon insertions in *lacZ*.

*E. coli* strain	*lacZ* allele	β‐Galactosidase activity (Miller units)	Relative activity^a^	Source
MET20	*lacZ* ^+^ (pMET20 vector)	19,000	1	Pedersen et al. (2019)
MET29	7 fast codons, Glu(GAA)‐rich	300	0.016	Pedersen et al. (2019)
MET30	14 fast codons, Glu(GAA)‐rich	130	0.0066	Pedersen et al. (2019)
MET31	21 fast codons, Glu(GAA)‐rich	50	0.0029	Pedersen et al. (2019)
BKG80	*lacZ* ^+^ (pMAS2 vector)	34,200	1	This study
BKG82	8 fast codons, Ala(GCU)‐rich	2800	0.082	This study
BKG84	Fastest synonymous codons, positions 1–30	900	0.026	This study

^a^ Activity relative to the corresponding wildtype reference strain (MET20 for the MET series; BKG80 for the BKG series).

We next assayed premature transcription termination in the new *lacZ* variants by RT‐qPCR. As shown in Figure 3b, the new variants with early fast‐translated codons, BKG82 and BKG84, lead to premature transcription termination of approximately the same magnitude as seen for MET30.

The prematurely terminating *lacZ* constructs are all enriched for codons expected to undergo rapid translation. For a codon to be translated fast, both the tRNA and the cognate amino acid must be present at concentrations that yield near‐saturating levels of the ternary aa‐tRNA‐GTP‐EFTu complex for binding to the ribosomal A‐site. Absolute in vivo translation rates have only been measured for a few codons (Sørensen and Pedersen 1991), and the same codon can be translated at different speeds dependent on the growth conditions and the sequence context (Pedersen 1984; Dong et al. 1996; Mohammad et al. 2019). To obtain a conservative list of the codons that are most likely to be translated rapidly under most conditions, we applied five filters.

First, we retained only the codons that are preferred in highly expressed genes (Sharp and Li 1987), reflecting strong evolutionary selection for translational efficiency. Second, we excluded codons for amino acids whose biosynthesis is regulated by attenuation because this regulatory mechanism responds to the external concentrations of the amino acids (Trp, His, Leu, Ile, Val, Thr, and Phe). Attenuation relies mechanistically on ribosome stalling at cognate codons under amino acid limitation, implying that charged tRNA levels for these amino acids fall below saturation in vivo. Third, we required that the cognate tRNA be encoded by multiple gene copies in the *E. coli* K‐12 genome (Dong et al. 1996), since tRNA abundance correlates with gene copy number and codons decoded by abundant tRNAs are more likely to experience saturating ternary complex delivery. Fourth, we excluded codons for amino acids that are not synthesized directly from central metabolic intermediates, reasoning that a lengthy biosynthetic pathway may limit amino acid availability and therefore reduce charged tRNA pools during rapid growth (Arg, Lys). Fifth, we excluded codons for proline, as the pyrrolidine ring of proline renders it a poor peptidyl transfer substrate both as a donor and acceptor in the peptidyl transferase center, causing intrinsic elongation slowdown independent of tRNA availability (Mohammad et al. 2019; Wohlgemuth et al. 2008; Pavlov et al. 2009). Codons passing all five filters (Glu codon GAA, Ala codon GCU, Asp codon GAC, Gln codon CAG, Ser codon UCU, Gly codon GGU, Asn codon AAC) were considered fast‐translated with high confidence, as both the aminacylated tRNA delivery step and the peptidyl transfer step are expected to proceed rapidly. We emphasize that this set of seven fast‐translated codons is our most conservative estimate. Other preferred codons are likely to be translated fast under many growth conditions.

We then examined the prevalence of our conservative set of fast‐translated codons in the first 30 codons of the genes used for Figure 2. Indeed, as shown in Figure 4a, the fast codons are generally more prevalent in the first 30 codons of the engineered *lacZ* variants than in the natural genes. However, several natural sequences were on par with pMET29, which had 8/30 fast codons by our restrictive estimate, suggesting that this number of fast codons alone does not lead to premature termination. As expected, rare codons were nearly absent from this set of highly expressed genes (Figure 4b). The inserts did not clearly separate from the natural genes in terms of the CAI of the first 30 codons, except pBKG84, which was specifically designed to reach the highest possible CAI for the *lacZ* N‐terminal peptide sequence (Figure 4c). We remark that the CAI of the earliest 30 codons was lower than the CAI of the whole protein coding sequence for all the natural genes analyzed (mean CAI difference 0.14 ± 0.09), in keeping with the hypothesis that there is a selective pressure for maintaining low‐CAI codons in the early part of coding sequences, even in highly expressed genes (Bulmer 1988; Tuller et al. 2010; Mitarai et al. 2008).

**FIGURE 4:**
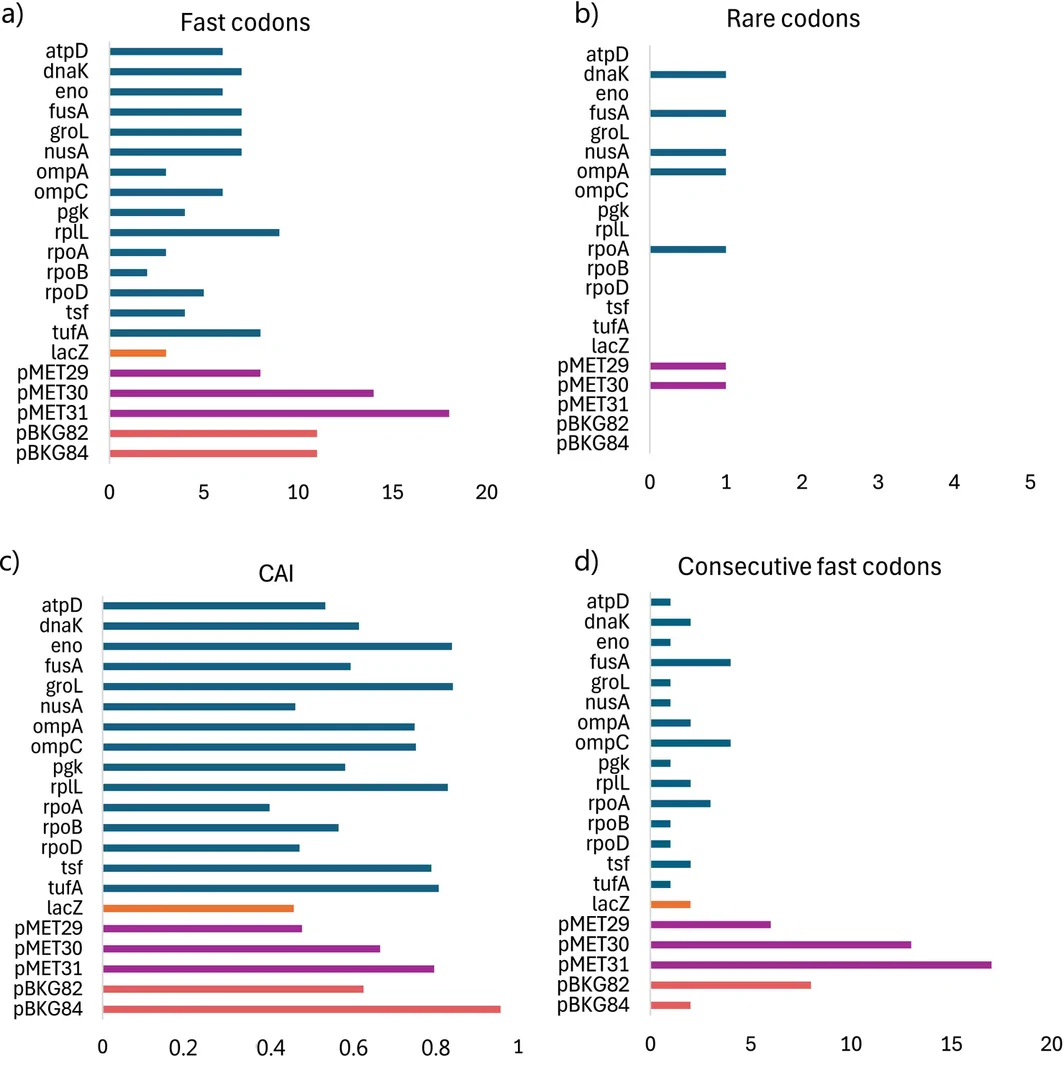
Prevalence of codons with specific properties in the initial 30‐codon stretch of the indicated genes. (a) Number of high‐confidence fast‐translated codons (AAC, CAG, GCU, GAA, GAC, GGU, UCU). (b) Number of rare codons (AGG, AGA, AUA, CUA, CGA, CCC). (c) CAI score of initial 30‐codon peptide. (d) Length of the longest run of high‐confidence consecutive fast codons (AAC, CAG, GCU, GAA, GAC, GGU, UCU).

Finally, we computed the longest run of consecutive fast codons found in the first 30 codons of the natural and synthetic genes. As shown in Figure 4d, the pMET29, pMET30, pMET31, and pBKG82 synthetic *lacZ* variants have longer runs of fast codons (≥ 6 codon stretch) than any of the natural genes (≤ 4 codon stretch). A genome‐wide search for consecutive runs of the seven high‐confidence fast‐translated codons confirmed the absence of such runs in the early regions of *E. coli* genes. No genes contained a run of six or more consecutive high‐confidence fast codons within the first 30 codons of the open reading frame, and only two genes (*ascB* and *yfgM*) contained a run of five. Synthetic *lacZ* variant pBKG84 showed premature transcription termination but did not contain a long run of the fast codons included in our list. Our restrictive list is not exhaustive, and more of the high‐CAI codons are likely fast‐translated under most growth conditions. Given that the early region of pBKG84‐*lacZ* was codon‐optimized from 1 to 30, we reason that the sequence is likely to contain one or more long runs of consecutive fast‐translated codons in the early region.

Based on this analysis, a run of consecutive fast codons is the feature that most clearly sets the prematurely terminating sequences apart from natural genes, and we argue therefore that the stretch of six consecutive fast codons may be causally linked to premature transcription termination.

To gain further insight into the location of the premature transcription termination sites, we studied the *lacZ* mRNAs by Northern blot analysis. Figure 5a shows a Northern blot of RNA from the induced MET20 strain expressing wildtype *lacZYA*, and the induced MET30 strain with an insert of fast codons early in *lacZ*. A radiolabeled reverse primer from the 5′ *lacZ* qPCR was used as the probe (see Figure 1a). In this experiment we also investigated whether speeding up translation by addition of amino acids to the minimal medium (Pedersen 1984) or speeding up transcription by the addition of uracil (Poulsen and Jensen 1987) had any influence on the termination sites. As seen in Figure 5a (compare lanes 1–6), the band pattern looked the same independent of growth supplements, and the six samples for each strain can therefore be regarded as replicates.

**FIGURE 5:**
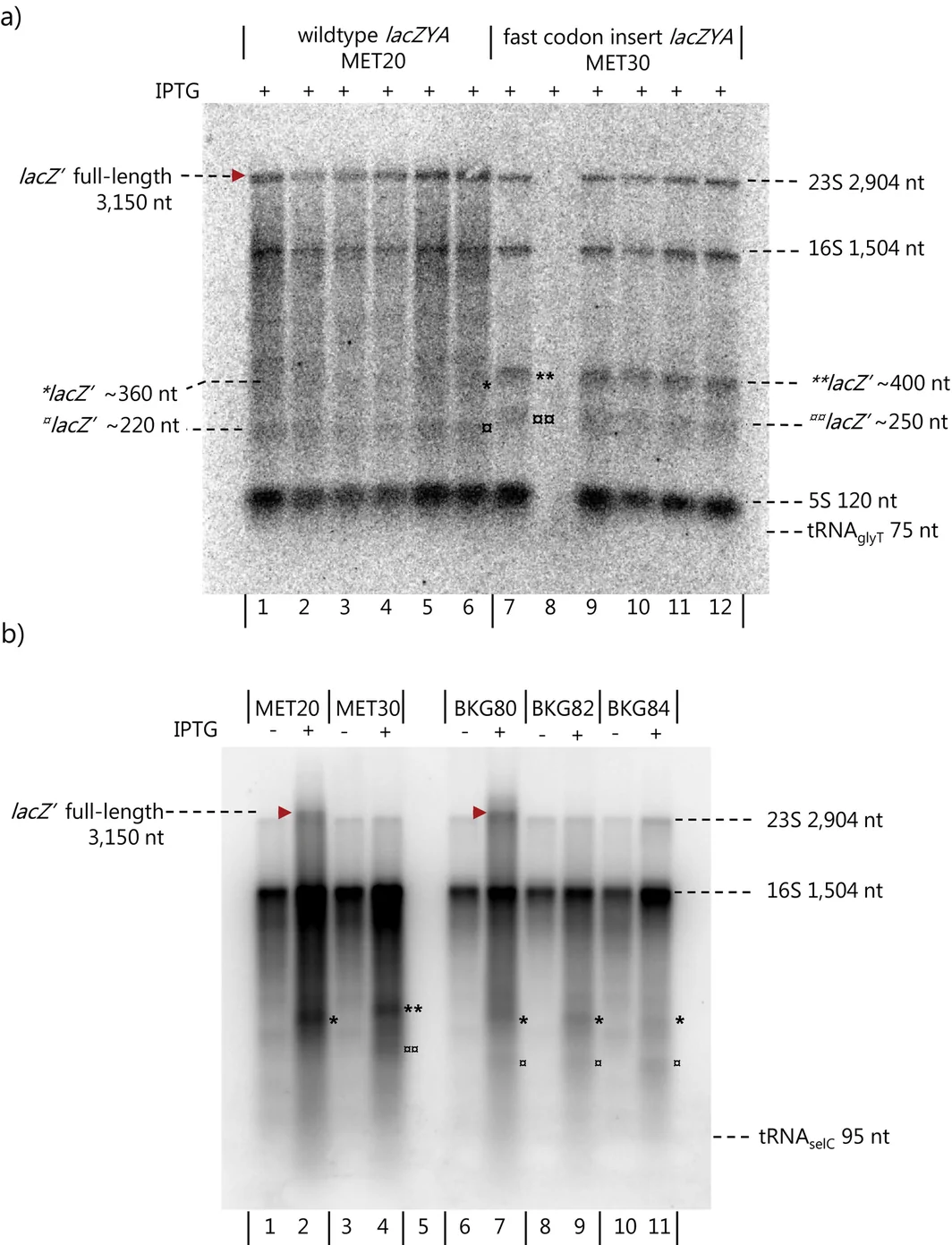
Distinct prematurely terminated RNAs in strains with fast‐translated codon inserts. (a) RNA from the fully induced wildtype strain MET20 (lanes 1–6) and from the fully induced strain MET30 with early fast translated codons (lanes 7–12) were analyzed on a 2% agarose, 6% formaldehyde gel using a ^32^P‐labeled reverse primer from the *5*′ *lacZ* qPCR primer set (see Figure 1a) as probe. Cultures of the indicated *E. coli* strains were grown in MOPS minimal medium with 0.2% proline as carbon source (see Section 4). Cultures were IPTG‐induced for 20 min in the presence of 5 mM cAMP before harvest. After 5 min of induction, the cultures were divided into three where one received no supplements, one was supplemented with 0.13 mg/mL of all 20 amino acids except methionine and lysine (Neidhardt et al. 1974), and one was supplemented with 3 μg/mL uracil. Lanes numbered from left to right contained 10 μg RNA from MET20 with no additions (lanes 1, 2); MET20 with amino acids added (lanes 3, 4); MET20 with uracil added (lanes 5, 6); MET30 with no additions (lanes 7,8 (lost sample)); MET30 with amino acids added (lanes 9, 10); MET30 with uracil added (lanes 11, 12). Size markers were 23S and tRNA^glyT^, which were probed separately; see Figure S2a. (b) RNA from uninduced and fully induced cultures of MET20 (lanes 1, 2), MET30 (lanes 3, 4), BKG80 (lanes 6, 7), BKG82 (lanes 8, 9), and BKG84 (lanes 10, 11) were analyzed on a 2% agarose, 6% formaldehyde gel using a ^32^P‐labeled reverse primer from the *5*′*lacZ v2* qPCR primer set (see Figure 3a) as probe. Lane 5 contains no sample. Cultures of the indicated *E. coli* strains were grown in MOPS minimal medium with 0.2% proline as carbon source (see Section 4) and induced with 1 mM IPTG for 40 min before harvest. Size markers were 23S and the spike‐in tRNA^selC^, which were probed separately; see Figure S3a. Symbols marking individual bands are used as in panel (a).

Despite the use of the 5′‐*lacZ*‐targeted probe, the most prominent bands seen in Figure 5a reflect probe binding to the stable 5S, 16S and 23S ribosomal RNA species that are present in huge excess of specific mRNAs. This hybridization issue was previously reported for 5′ *lacZ* probes (Murakawa et al. 1991). We confirmed the identity of one of these bands by probing the same membrane separately for 23S rRNA and further probed for tRNA^glyT^ as an additional size marker (see Figure S2a). The nascent *lacZYA* transcript is processed to yield a predominant transcript encoding full‐length *lacZ* of 3150 nt (Murakawa et al. 1991). This full‐length *lacZ* mRNA was detected with the *5*′ *lacZ* probe immediately above the 23S rRNA band in all six samples of MET20, whereas it could not be detected in any of the samples of MET30 RNA (Figure 5a red arrowhead, Figure S2b).

We repeated the experiment with more *lacZ* variants and another early‐*lacZ* probe, namely the reverse primer of the *5*′ *lacZ v2* primer set, which is complementary to the *5*′ *lacZ* region shared by all the constructs (Figure 5b). This probe (and any other *lacZ*‐specific probe we tested, data not shown) also yielded prominent bands at the positions of ribosomal RNAs, although in different proportions than the probe used for Figure 5a. Similarly to Figure 5a, however, we detected full‐length *lacZ* mRNA transcript in IPTG‐induced cultures expressing the wildtype *lacZ* mRNA (MET20 and BKG80), while the full‐length *lacZ* mRNA could not be detected in the IPTG‐induced cultures expressing any of the fast‐translated variants (MET30, BKG82, BKG84), or in any of the uninduced cultures (see Figure 5b, red arrowhead). The absence of full‐length *lacZ* mRNA in variants that undergo fast translation of the early *lacZ* mRNA region is consistent with premature transcription termination of the majority of *lacZ* transcripts and strongly reduced protein expression.

In further support of premature transcription termination, several incomplete *lacZ* transcripts were detected by the *5*′ *lacZ* probes, particularly in the 200–400 nt region of the blots. Specifically, in Figure 5a, faint bands at ~220 nt (marked by ¤) and ~360 nt (marked by *) were present in the MET20 strain encoding wildtype *lacZ*, which corresponded to prominent bands at ~250 nt (marked by ¤¤) and ~400 nt (marked by **) in the MET30 strain. The 39 nt insert of fast early codons in MET30 is responsible for the increased band sizes compared to wildtype *lacZ* in MET20. Figure 5b shows that the same ~360/400 nt band could be detected in RNA from MET20/MET30 with the alternative *5*′ *lacZ v2* probe, while the shorter ~220 nt band was indiscernible in MET20, but visible as a faint ~250 nt band in MET30. Figure 5b further shows faint bands at ~220 nt and ~360 nt in the fast‐early‐codons *lacZ* variants encoded by BKG82 and BKG84, which have the same length as wildtype *lacZ* mRNA since the fast codons in these constructs were obtained by sequence replacement rather than sequence insertion.

The *lacZ* gene has been thoroughly characterized in terms of intragenic transcription termination sites (Ruteshouser and Richardson 1989). Specifically, in vitro RNA synthesis terminated at Rho‐dependent intragenic terminators at positions 180 (8%), 220 (13%), 379 (30%) and 463 (56%) from the 5′ end of the *lacZ* wildtype transcript. The 3′ endpoints at 379 and 463 nt were also observed in vivo from *lacZ* mRNA carrying a nonsense mutation at codon 18, and the 220 nt 3′ endpoint was observed in vivo after amino acid starvation by serine hydroxamate treatment, suggesting that these termination sites become physiologically relevant during transcription of *lacZ* in vivo when the ribosome lags behind RNA polymerase and the newly transcribed RNA is not being co‐translated (Ruteshouser and Richardson 1989). The prominent bands of approximately 220(+insert) and 360(+insert) seen in Figure 5 might well reflect transcription termination at the reported 220 and 379 nt Rho‐dependent termination sites. We note that these sites are far downstream from the fast codons. For example, the insert of early fast codons stretches from position +71 to +92 in the *lacZ* mRNA from MET30, while the prominent bands correspond to termination events much further into the RNA, at approximately +250 and +400.

In summary, our data show that a stretch of consecutive fast translated codons early in the *lacZ* gene results in premature transcription termination up to several 100 bases downstream of the fast codons.

## 3 Discussion and Perspectives

Decoupling of RNA polymerase and the leading ribosome is the best‐characterized mechanism by which a translation event can lead to transcription termination. It is therefore a likely assumption that the unusual termination events we observe here involve loss of RNA polymerase‐ribosome coupling at some point during or after translation of the consecutive fast codons, exposing downstream mRNA to Rho and leading to termination at the next available Rho utilization site. We showed previously that an insert of 36 fast‐translated GAA codons *late* in the *lacZ* gene has no effect on gene expression but the same fast‐translated insert leads to transcription termination if located in the first approximately 30 codons (Pedersen et al. 2011), which corresponds closely to the region of selection for non‐biased codons in natural genes (Bulmer 1988; Tuller et al. 2010).

To explain these findings, we propose the following mechanism based on the kinetic and physical mechanics of translation. First, an analogy to threading a needle. It is common knowledge that it is important not to push the thread too fast into the needle eye because the thread then bulges out, blocks the eye and one must start over. The thread must first pass through the eye; then one can grab it and pull it out fast. Analogously, for translation initiation, we suggest that it is important not to synthesize the peptide faster than it can enter and pass through the exit tunnel. If early translation occurs too fast, translation may continue, but the peptide would bulge out before or inside the peptide exit tunnel. Eventually, bulging would create a mechanical conflict that stalls or disrupts the ribosome, after which RNA polymerase would terminate at a downstream Rho‐dependent termination site.

Interspersed slowly translated codons might give the N‐terminal peptide time to diffuse and reorient in the tunnel and prevent the bulging from reaching a size where the bulge becomes irreversible. Once the first part of the nascent peptide is clear of the exit tunnel, chaperones will assist it, folded domains will form and there will no longer be a need for slow translation. We note that the estimated length of the ribosome exit tunnel could accommodate approximately 30 amino acids (Bui and Hoang 2020). Consistent with our proposal, coarse‐grained simulations of nascent chain escape indicate that the exit tunnel can itself act as a kinetic trap, with escape kinetics depending strongly on tunnel shape (Bui and Hoang 2020). A bias toward fast‐translated codons after about codon 30, as observed for genes encoding highly expressed proteins, would then optimize ribosome translation capacity without the risk of jamming the exit tunnel, which would be highly significant for cell fitness, especially at high growth rates. Our proposed mechanism is a working model that awaits experimental verification but is testable for instance by cryo‐electron microscopy. While the mechanism we envisage differs from that of well‐characterized stalling peptides, which arrest an already‐threaded nascent chain through sequence‐specific contacts with the tunnel walls that propagate to the peptidyl transferase center (Nakatogawa and Ito 2002; Gong and Yanofsky 2002; Su et al. 2017), this body of work establishes the relevant precedent that the exit tunnel is a site at which translation can be impeded. Our hypothesis extends this principle in two respects. We propose that the stalling arises from a failure of the nascent peptide to enter the tunnel correctly rather than from arrest within it, and that it is driven by translation kinetics alone, independent of the peptide sequence. The broader observation that elongation speed influences co‐translational folding of the nascent chain (Komar 2009; Yu et al. 2015) lends further plausibility to the idea that early elongation kinetics could affect how the nascent peptide negotiates the entrance to, and passage through, the exit tunnel.

Our model invokes a stalled leading ribosome within or immediately downstream of the fast‐codon region, and trailing ribosomes would be expected to queue behind it. SmrB‐mediated mRNA cleavage at collided ribosomes (Saito et al. 2022) should therefore be considered as an alternative origin of truncated transcripts. However, the prominent truncated species we detect map up to several 100 nucleotides downstream of the inserts, at positions coinciding with intragenic Rho‐dependent termination sites (Ruteshouser and Richardson 1989), a coincidence for which collision‐triggered cleavage offers no explanation. We therefore consider endonucleolytic cleavage at collided ribosomes an unlikely primary source of the transcripts we observe, although we cannot exclude that it contributes.

We next sought to characterize the proposed termination mechanism more quantitatively. Our model posits that premature termination occurs when the early region of an open reading frame is translated faster than some threshold, such that the nascent peptide bulges rather than threading the exit tunnel. Because of the stochastic nature of the biochemical events in translation, individual codon translation times vary, and even an insert of uniformly fast codons will, on a fraction of transcripts, happen to be translated slower than the threshold and escape termination. The magnitude of the escaping fraction therefore reports on where the threshold lies relative to the translation‐time distribution.

To model this, we treated translation of each codon as a single‐step process with a constant rate, using the average rate of 22 codons/s previously determined for Glu codon GAA as the fast rate and 6 codons/s as the slow rate (Sørensen and Pedersen 1991). The shortest insert that gives strong premature termination (strain MET29) contains an uninterrupted six‐codon stretch of fast codons (Table 2, Section 4) and terminates about 82% of transcripts prematurely (Pedersen et al. 2019). We computed the distribution of times required to translate these six fast codons (Figure 6a, red curve; calculation in Section 4). Equating the 18% of transcripts that escape termination with the slow tail from this distribution places the threshold rate at approximately 0.4 s to translate the six‐codon stretch (Figure 6a, dotted vertical line).

**TABLE 2:** RNA sequence of the early coding region of *lacZ* variants used in this study.

Plasmid	Sequence of *lacZ* early coding region
pMET20 and pBKG80 Codon 1–30	AUG ACC AUG AUU ACG GAU UCA CUG GCC GUC GUU UUA CAA CGU CGU GAC UGG GAA **AAC** CCU GGC GUU ACC CAA CUU AAU CGC CUU GCA GCA
pMET29 Codon 1–37	AUG ACC AUG AUU ACG GAU UCA CUG GCC GUC GUU ggu cac (gaa)_2_ gcu (gaa)_2_ ggu cac (cgu)_2_ agg cgg gga **AAC** CCU GGC GUU ACC CAA CUU AAU CGC CUU GCA GCA
pMET30 Codon 1–44	AUG ACC AUG AUU ACG GAU UCA CUG GCC GUC GUU ggu cac (gaa)_3_ gcu (gaa)_4_ gcu (gaa)_3_ ggu cac (cgu)_2_ agg cgg gga **AAC** CCU GGC GUU ACC CAA CUU AAU CGC CUU GCA GCA
pMET31 Codon 1–51	AUG ACC AUG AUU ACG GAU UCA CUG GCC GUC GUU ggu cac (gaa)_3_ gcu (gaa)_4_ gcu (gaa)_4_ gcu (gaa)_5_ ggu cac (cgu)_2_ agg cgg gga **AAC** CCU GGC GUU ACC CAA CUU AAU CGC CUU GCA GCA
pBKG82 Codon 1–30	AUG ACC AUG AUU acc gac (gcu)_7_ CGU CGU GAC UGG GAA **AAC** CCU GGC GUU ACC CAA CUU AAU CGC CUU GCA GCA
pBKG84 Codon 1–30	AUG ACC AUG auc acc gac ucu CUG gcu guu GUU cug cag CGU CGU GAC UGG GAA **AAC** ccg GGC GUU ACC cag cug aac CGC cug gcu

*Note:* Lowercase codons indicate differences from the wildtype sequence. Codons in brackets are repeated as indicated by the subscript number. Codon 19 of the wildtype sequence is bold to ease the comparison between sequences. Codons on our high‐confidence list of fast codons are shown in green font.

**FIGURE 6:**
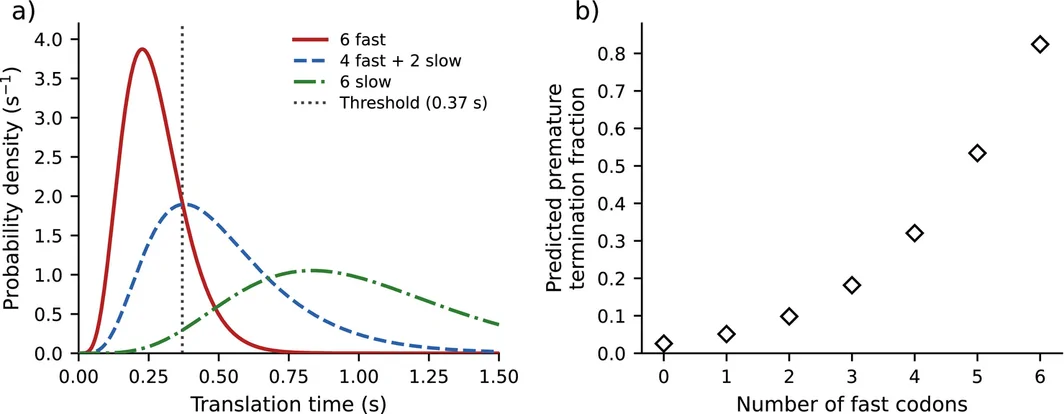
Predicted time distribution for translation of a six‐codon window assuming a stochastic translation mechanism. (a) Red curve shows six codons translated at 22 codons/s; blue curve shows four codons at 22 codons/s and two codons at 6 codons/s; green curve shows six codons at 6 codons/s. Dotted line marks the threshold translation time of 0.37 s, obtained by equating the 18% of MET29 transcripts that escape termination with the slow tail of the six‐fast‐codon distribution (red curve). (b) Fraction of transcripts predicted to terminate transcription prematurely as a function of the number of fast‐translated codons in the six‐codon window relevant for threading the ribosome exit tunnel. The ordinate value showing premature termination for six slowly translated codons is 0.026.

Having calibrated the threshold, we asked how the predicted transcription termination frequency depends on the number of fast codons in a six‐codon window. For comparison, we also calculated the time distribution for four fast and two slowly translated codons (Figure 6a, blue curve) and six slowly translated codons (Figure 6a, green curve). We then estimated, for six‐codon stretches with progressively more of the fast codons, the fraction of translation times that are shorter than the threshold and thus should give rise to premature transcription termination according to our model (Figure 6b).

Many natural genes contain several fast‐translated codons within an early six‐codon stretch. Our model predicts several additional percent of transcripts will terminate prematurely per additional fast codon in this window, above a baseline of 2.6% predicted termination even for an all‐slow six‐codon window. This seems a very high number, considering that selection could reduce the excess wasteful termination simply by changing fast‐translated codons to slower‐translated synonymous codons. The discrepancy suggests that the absolute translation rates(Sørensen and Pedersen 1991) used in our single‐step model may differ from codons occurring in natural sequences. A plausible resolution is that codon translation rate depends on sequence context. The fast translation rate measured for Glu codon GAA may apply only when its neighbor codons also pair with the anticodon without wobbling, as was the case in the constructs used to measure it (Sørensen and Pedersen 1991). A codon such as GAG, which has wobbling codon‐anticodon interactions, would be translated more slowly and would also slow its neighbors. Mechanistically, when three non‐wobbling codons occupy the ribosomal A, P and E sites, their codon‐anticodon could form a continuous base stack of up to nine base pairs, increasing the affinity for the incoming aminoacyl‐tRNA and accelerating accommodation (Hughes and Chevance 2018; Chevance and Hughes 2017). This model is not in contradiction to our previous results. The in vivo translation rate for GAA (Sørensen and Pedersen 1991) was performed on sequences with consecutive identical fast‐translated codons and our finding that highly expressed genes were translated twice as fast as genes using the non‐biased codons (Pedersen 1984) could be because the highly expressed genes have a high preponderance of non‐wobbling codons ensuring that almost all codons have a non‐wobbling neighbor codon and thus an increased translation rate. Our results thus support the hypothesis of the ribosome reading a “triplet of triplets” genetic code (Hughes and Chevance 2018; Chevance and Hughes 2017).

Finally, we note that we detected transcription termination caused by the inserts studied here only because the effect is dramatic. Owing to the stochastic nature of translation, it is likely that even a few fast‐translated codons occasionally produce a local translation rate high enough to cause peptide bulging and therefore some wasteful transcription termination. Such low‐level termination would be undetectable with our present methods but would nonetheless be important for cell economy. We therefore argue that avoidance of premature termination is an important contributing reason for the elevated presence of slowly translated codons in the early part of bacterial genes (Bulmer 1988; Tuller et al. 2010; Eyre‐Walker and Bulmer 1993), acting alongside previously proposed functions such as minimizing ribosome queuing on the downstream mRNA (Tuller et al. 2010; Mitarai et al. 2008). Importantly, premature transcription termination is a consequence only in bacteria such as *E. coli* , where transcription and translation are physically coupled, but unsuccessful threading of the ribosome exit tunnel would be detrimental for protein synthesis efficiency in all cells and therefore to be avoided also in eukaryotic cells.

## 4 Experimental Procedures

### 4.1 Bacterial Strains and Growth Conditions

The *E. coli* strains and growth conditions used in this work were described previously (Pedersen et al. 2019). Briefly, cultures of *E. coli* NF1830 were cultured to balanced growth in morpholinepropanesulfonic acid (MOPS) minimal medium (Neidhardt et al. 1974) with 0.2% glycerol as carbon source supplemented with 10 mg/mL isoleucine, 10 mg/mL valine, and 20 mg/mL leucine or 0.2% proline as carbon source supplemented with 20 mg/mL leucine, as indicated in the legend to each figure. The *lac* operon was induced by addition of 1 mM IPTG for 15–40 min, as indicated.

The MET20 strain is *E. coli* NF1830 harboring pMET20, a plasmid derivative of pBR322 carrying the complete *lac* operon (Pedersen et al. 2019). The strains MET29, MET30, and MET31 carry variants of this plasmid with inserts of fast translated codon sequences at codon 13 of *lacZ* in the plasmid. BKG80 harbors the pMAS2 plasmid, carrying the wildtype *lac* operon from the EcoRI site at position −410 before the *lacZ* gene to position +3334 (the *AvaI* site in *lacY*) in the same plasmid backbone as pMET20 (Sørensen et al. 1989). BKG82 and BKG84 carry pMAS2 with sequence modifications in the early part of the *lacZ* coding sequence. Table 2 shows the sequence of the early part of *lacZ* in the five plasmids.

### 4.2 Plasmids Constructed in This Study

Plasmid pBKG82 was constructed by In‐Fusion Cloning (Takara Biotech) with overlapping primers containing the desired substitutions:

Primer lacZ hydfob+ Fw: CGCTGCTGCTGCTGCTGCTGCTCGTCGTGACTGGGAAAACC.

Primer lacZ hydfob+ Rv: GCAGCAGCAGCAGCGTCGGTAATCATGGTCATAGCTGTTTCC.

Similarly, pBKG84 was constructed with In‐Fusion Cloning (Takara Biotech) by replacing part of the *lacZ* gene with a gBlock (Integrated DNA Technologies) containing the desired substitutions using the following DNA primers and fragment:

lacZ fast Fw2: CCTTTCGCCAGCTGGCGTAATAG.

lacZ fast Rv2: TTTCCTGTGTGAAATTGTTATCCGCTCAC.

IDT gBlock: AATTTCACACAGGAAACAGCTATGACCATGATTACGGATTCACTGGCCGTCGTTTTACAACGTCGTGACTGGGAAAACCCTGGCGTTACCCAACTTAATCGCCTTGCAGCACATCCCCCTTTCGCCAGCTGG.

### 4.3 RNA Purification

RNA was extracted by the hot phenol method, including the use of a whole‐cell spike‐in culture overexpressing the rare tRNA^selC^ for normalization purposes in Northern blots (Prossliner et al. 2023). RNA for quantitative PCR was further processed with ApaLI restriction enzyme (ThermoFisher Scientific) to digest residual plasmid DNA prior to DNase treatment with TURBO DNA‐free Kit (Invitrogen) to deplete remaining genomic and plasmid DNA.

### 4.4 Reverse Transcriptase Quantitative PCR

First‐strand complementary DNA (cDNA) was reverse transcribed from ≈150 ng of total RNA with Thermo Scientific RevertAid RT Kit (#K1691) using the gene specific reverse primer from each qPCR primer pair shown in the list below.

1/10 of the total synthesized cDNA was combined with SsoAdvanced Universal SYBR Green Supermix (Bio‐Rad) and analyzed in triplicate by qRT‐PCR using the QuantStudio 3 system (Applied Biosystems). The relative level of RNA is calculated as the signal ratio between the target transcript and the reference gene *cysG*, using the $2^{-\Delta \Delta C_{t}}$ method (Livak and Schmittgen 2001).

For RT‐qPCR, we used the following primer sets:

1. *lacZ 5*′: Amplifies the part of the *lacZ* mRNA upstream of the inserted fast codons in MET 29, 30, and 31 in addition to detecting the wildtype *lacZ* mRNA from mRNA start to codon 11. Forward primer: 5′AATTGTGAGCGGATAACAATTTCACACAG. Reverse primer: 5′ AACGACGGCCAGTGAATCCGT.
2. *lacZ 5*′ *v2*: Amplifies the part of *lacZ* mRNA upstream of the inserted fast codons in all 5 constructs (MET29, 30, 31 and BKG82, 84) including the two wildtype *lacZ* mRNA in MET20 and BKG80 from mRNA start to codon 3. Forward primer: 5′AATTGTGAGCGGATAACAAT. Reverse primer: 5′ CATGGTCATAGCTGTTTC.
3. *lacZ* 500: Amplifies *lacZ* mRNA in the region from codon 167–199. Forward primer: 5′TTTGCCGTCTGAATTTGACCTGAGCG. Reverse primer: 5′ TCTTCCAGATAACTGCCGTCACTCCAG.
4. *lacZ 3*′:Amplifies the 3′ end of *lacZ* mRNA from codon 967 to 1000. Forward primer: 5′ ACTGATGGAAACCAGCCATCGCCA. Reverse primer: 5′ CCAGGAGTCGTCGCCACCAA.
5. *lacA*: Amplifies *lacA* mRNA from codon 18 to codon 64. Forward primer: 5′ GTGCGAAGGCTTACCGGAAAAAAGACTTC. Reverse primer: 5′ CTACCCAGGCGTTTTCCCCTACC.
6. *cysG*: Amplifies the *cysG* mRNA to be used for normalization. Forward primer: 5′ TTGTCGGCGGTGGTGATGTC. Reverse primer: 5′ ATG CGG TGA ACT GTG GAA TAA ACG.

### 4.5 Northern Blot

Northern blots were performed essentially as described (Prossliner et al. 2023).

*lacZ* 5′ probe: 5′‐CATGGTCATAGCTGTTTCCTG.

*lacZ* 5′ v2 probe: 5′‐CATGGTCATAGCTGTTTC.

23S rRNA probe: 5′‐TATCAGCCTGTTATCCCCGG.

tRNA^glyT^ probe: 5′‐CCCGCATCATCAGCTTGGAAGGC.

tRNA^selC^ probe: 5′‐ATTTGAAGTCCAGCCGCC.

### 4.6 β‐Galactosidase Assays

Cultures of BKG80, BKG82, and BKG84 were grown in MOPS minimal medium supplemented with 0.2% glycerol, 10 mg/mL isoleucine, 10 mg/mL valine, and 20 mg/mL leucine. Samples were harvested immediately before and 10, 20, 30, and 40 min after induction with 1 mM IPTG. As in Pedersen et al. 2019 (Pedersen et al. 2019), cells were opened by sonic treatment, and the β‐galactosidase activities were measured essentially as described (Miller 1972). Miller units in Table 1 give the enzyme activity per cell mass for a fully induced culture.

### 4.7 Enumeration of Codon Types and Calculation of CAI Scores

The nucleotide sequences used in Figures 2 and 4 were obtained from the EcoCyc database (Keseler et al. 2021). The codon adaptation indexes for the N‐terminal peptides and the full protein sequences were calculated using the CAI tool of the EMBOSS software package and the standard EMBOSS data file Eecoli_high.cut, which is a table of codon usage in highly expressed *E. coli* proteins (Rice et al. 2000).

### 4.8 Analysis of Gene Sequences to Find Consecutive Fast Codons

The data of the gene sequences of MG1655 was obtained from the BioCyc database in smarttable ([EcoCyc21]; Keseler et al. 2021). The analysis was done by using a python code developed by the authors. The code and the data file are available at the following link: https://sid.erda.dk/cgi‐sid/ls.py?share_id=gY0iCePbss.

### 4.9 Calculation of the Time Distribution for Translating a Codon Sequence at a Certain Speed

We assume that the translation of one codon is either fast at a rate *F* = 22 codons/s, or slow at a rate *S* = 6 codons/s (Sørensen and Pedersen 1991), and happens as a one‐step constant rate process. We further ignore any interaction between ribosomes. The distribution of the duration τ to translate a fast codon is then given by $P_{F}\left(\tau \right)=Fe^{-\mathrm{F\tau}}$. The distribution of duration to translate k fast codons is then given by

$$\begin{array}{c} P_{\mathrm{kF}}\left(\tau \right)=F^{k}\int_{0}^{\tau }dt_{1}\int_{0}^{\tau -t_{1}}dt_{2}\cdots \\ \;\int_{0}^{\tau -\sum_{j=1}^{k-2}t_{j}}dt_{k-1}e^{-F\left(\tau -\sum_{i=1}^{k-1}t_{i}\right)}\prod_{j=1}^{k-1}e^{-Ft_{j}}=\frac{F^{k}\tau^{k-1}}{\left(k-1\right)!}e^{-\mathrm{F\tau}}. \end{array}$$

Similarly, the duration distribution for translation of m slow codons is $P_{\mathrm{mS}}\left(\tau \right)=\frac{S^{m}\tau^{m-1}}{\left(m-1\right)!}e^{-\mathrm{S\tau}}$.

The rate of translating k fast codons and m slow codons is then $P_{\mathrm{kF},\mathrm{mS}}\left(\tau \right)=\int_{0}^{\tau }dtP_{\mathrm{kF}}\left(t\right)P_{\mathrm{kS}}\left(\tau -t\right)$. We perfomed the final integral using Maple release 2023.2 (Maplesoft, a division of Waterloo Maple Inc., Waterloo, Ontario) and plotted the obtained function in Figure 6a.

## Author Contributions

**Steen Pedersen:** conceptualization, investigation, funding acquisition, writing – original draft, methodology, validation, visualization, writing – review and editing, supervision. **Alberte Honoré Jepsen:** investigation, writing – original draft. **Kim Sneppen:** conceptualization, formal analysis. **Sine Lo Svenningsen:** conceptualization, funding acquisition, investigation, writing – original draft, writing – review and editing, visualization, project administration, supervision. **Bertil Gummesson:** investigation, writing – review and editing, visualization, methodology. **Namiko Mitarai:** software, methodology, investigation, writing – review and editing, conceptualization, visualization.

## Funding

This work was supported by Danmarks Frie Forskningsfond (8049‐00071B, 8021‐00280A).

## Ethics Statement

This study did not involve human participants, human material, or animals, and no ethical approval was required.

## Conflicts of Interest

The authors declare no conflicts of interest.

## Supporting information


**Table S1:** The amount of mRNA was determined by RT‐qPCR using the probes for either *lacA* or *lacZ 5*′ as in Figure 1 and described in Experimental Procedures. Triplicate samples were analyzed from either induced or uninduced cultures of the wildtype construct MET20 and the strain MET29 with 7 fast codons inserted into codon 13 of the *lacZ* gene. Induction ratio denotes the induced mRNA levels divided by the uninduced mRNA levels.
**Figure S1:** (a) Schematic of the *lacZYA* operon, illustrating the location of the primer sets used for RT‐qPCR. Symbols as in Figure 1a of the main text. (b) Quantification of the levels of mRNA from *lacZ 5*′ (gray bars), *lacZ 500* (blue bars), *lacZ 3*′ (red bars) and *lacA* (yellow bars) by RT‐qPCR on total RNA from MET20 (wildtype), MET29 (7 fast), MET30 (14 fast), MET31 (21 fast). mRNA levels were normalized to the *cysG* reference gene and are shown relative to wildtype levels.
**Figure S2:** Size markers for northern blot shown in Figure 5a.
**Figure S3:** Size markers for the northern blot shown in Figure 5b.
**Data S1:** Reference list.

## Data Availability

All data supporting the findings of this study are available within the article and its Supporting Information S1. Bacterial strains and plasmids generated in this study are available from the corresponding authors on reasonable request.
