# Supplementary material for: Rapid Translation of the Early Coding Region Promotes Premature Transcription Termination, Reducing Protein Expression in Escherichia coli
Source: Mol Microbiol. 2026 Sep 11;126(5):519–31. doi: 10.1111/mmi.70113 (PMC13725320; doi:10.1111/mmi.70113)
Supplement: Supplementary file 1 [file MMI-126-519-s001.pdf]

Supplementary material for

Rapid translation of the early coding region promotes premature transcription termination, reducing protein expression in *Escherichia coli*

by
Steen Pedersen[*], Alberte Honoré Jepsen, Bertil Kent Gummesson, Namiko Mitarai, Kim Sneppen, Sine Lo Svenningsen[*].

* Corresponding Authors
steenp@fastmail.com
sls@bio.ku.dk

Supplementary Text:

The RT-qPCR shown in Figure 1 was also performed with a *lacA*-specific primer set (see Suppl. Fig. S1). In the induced strains with inserts of fast translated codons, we found previously that the inserts caused a substantial reduction in the amount of induced LacA enzyme (1). As discussed in the main text, we found an up to twenty-fold reduction of the distal part of the *lacZ* mRNA in the strains with inserted fast translated codons (Fig. 1b). The *lacA* mRNA, however, was only reduced about two-fold in the strains with fast codon inserts in *lacZ* relative to wildtype. Furthermore, *lacA* mRNA levels did not vary with the length of the insert (Suppl. Fig. S1b).
To understand the unexpected high concentration of *lacA* mRNA in the prematurely terminating constructs, we also compared the amount of 5' *lac*Z and *lacA* mRNA from uninduced cultures to those from the fully induced cultures of the wild type MET20 and the strain MET29 with seven fast codons inserted into codon 13 of the *lacZ* gene. As seen in Suppl. Table S1, the degree of induction of the *lacA* part of the operon is approximately four-fold lower than that of the early part of the *lacZ* gene.

***E. coli* strain**	**induction ratio *lac*A**	**induction ratio *lacZ 5'***
MET20	13.9	78.8
MET29	18.4	55.7

Table S1: The amount of mRNA was determined by RT-qPCR using the probes for either *lacA* or *lacZ 5'* as in Fig. 1 and described in Methods. Triplicate samples were analyzed from either induced or uninduced cultures of the wildtype construct MET20 and the strain MET29 with 7 fast codons inserted into codon 13 of the *lacZ* gene. Induction ratio denotes the induced mRNA levels divided by the uninduced mRNA levels.

This indicates that *lacA* and possibly also *lacY* are transcribed from one or more additional promoters with a collective strength of about four times that of the uninduced *lac* operon promoter. Three transcription start sites were previously identified in this region by differential RNA sequencing (2). The activity of these additional promoters would obscure the effect of premature transcription termination occurring upstream of *lacA.* By contrast, to the best of our knowledge no additional transcription start sites have been detected internally to the *lacZ* coding sequence. Differences in mRNA levels internal to the *lacZ* ORF can therefore reasonably be interpreted to be due to transcription termination events, and we focus on those in the main text. We note that this calculation of relative promoter strength assumes the same halflife of the *lacZ* and *lacYA* mRNAs, which are separated during mRNA processing (3, 4). Thus, *lacA* mRNA levels reflect not only read-through transcription from the lac operon promoter but also constitutive transcription and potentially differential stability of the processed *lacYA* mRNA, making *lacA* mRNA levels a qualitative rather than quantitative indicator of premature termination within *lacZ*.

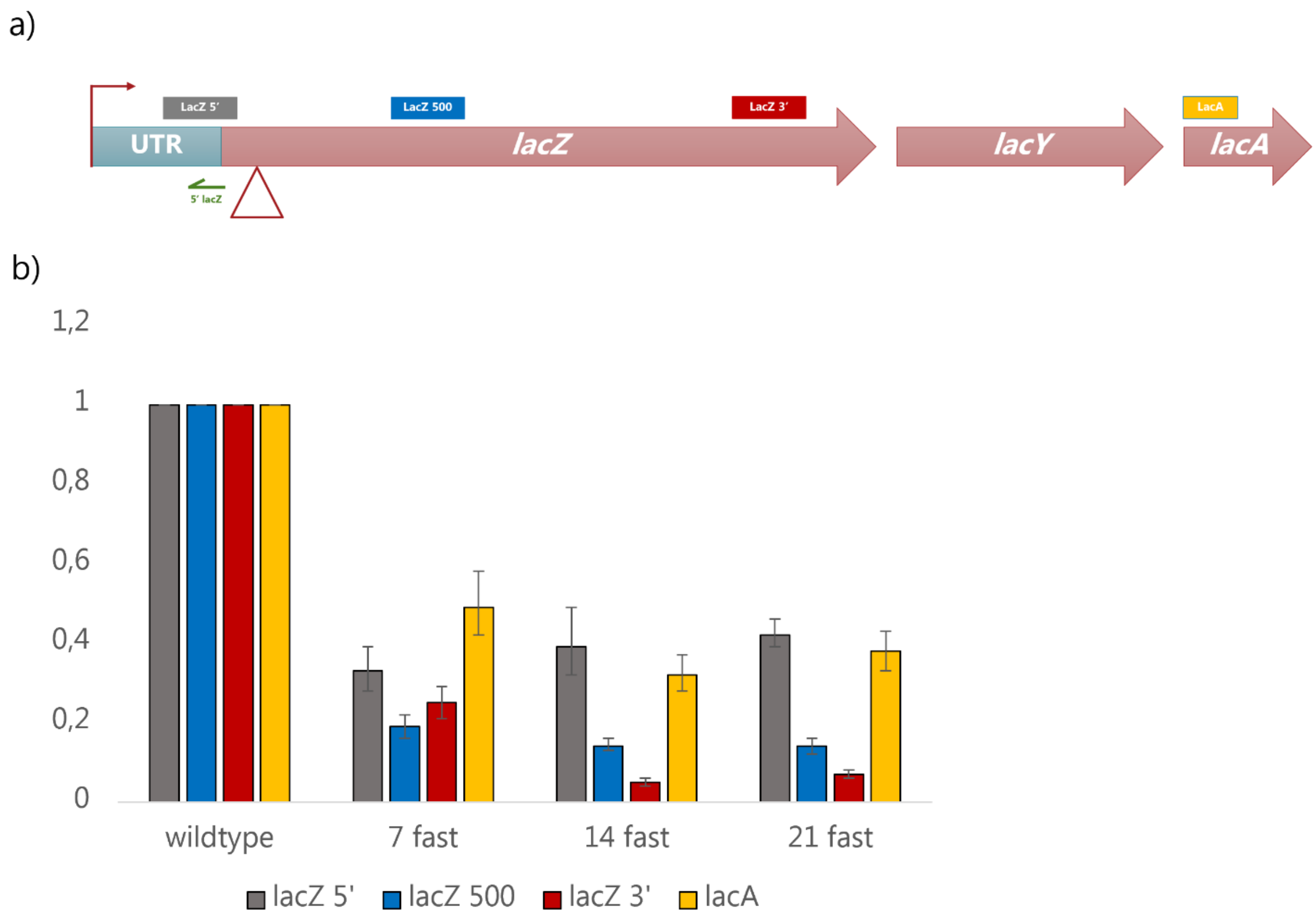

Supplementary Fig. S1. a) Schematic of the *lacZYA* operon, illustrating the location of the primer sets used for RT-qPCR. Symbols as in Fig. 1a of the main text. **b)** Quantification of the levels of mRNA from *lacZ 5'* (gray bars), *lacZ 500* (blue bars), *lacZ 3'* (red bars) and *lacA* (yellow bars) by RT-qPCR on total RNA from MET20 (wildtype), MET29 (7 fast), MET30 (14 fast), MET31 (21 fast). mRNA levels were normalized to the *cysG* reference gene and are shown relative to wildtype levels.

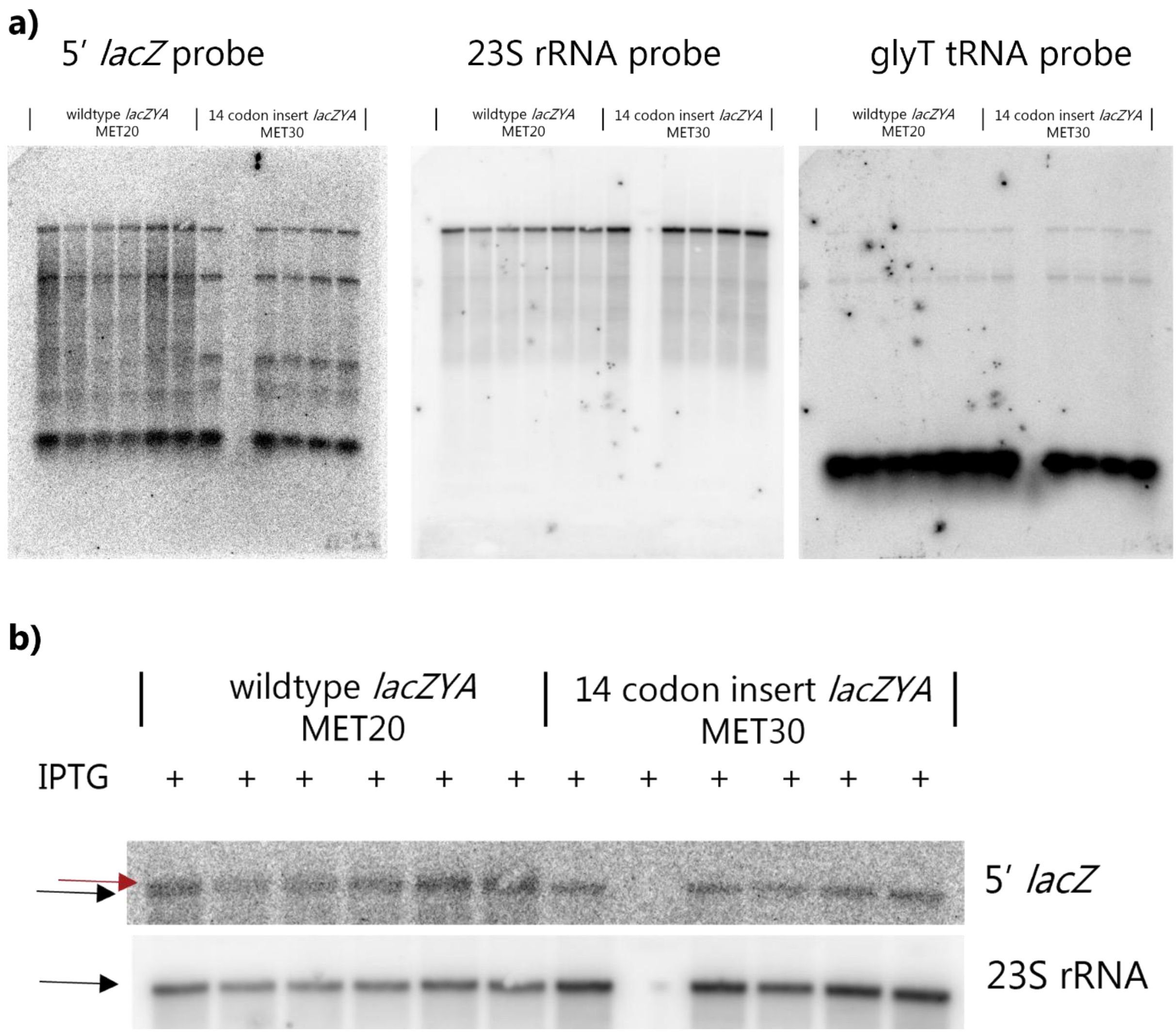

Supplementary Figure S2: Size markers for northern blot shown in Fig 5a.

a) The northern blot shown in Fig. 5a of the main text was independently probed for 23S rRNA and $tRNA^{glyT}$ to confirm the identity of the ribosomal RNA bands seen with the *5′ lacZ* probe and to add the tRNA size marker. **b)** Magnified section of the northern blots shown in panel A shows the presence of a double band in strain MET20 indicating the presence of both 23S rRNA (black arrow) and full-length *lacZ* mRNA (red arrow) when the membrane was probed with the *5′ lacZ*-specific probe (top). The full-length *lacZ* band was visible for all six replicates of MET20 cultures but could not be detected in the five replicate cultures of MET30. For all the samples, only the 23S rRNA band appeared when the membrane was probed with a 23S rRNA-specific probe (bottom).

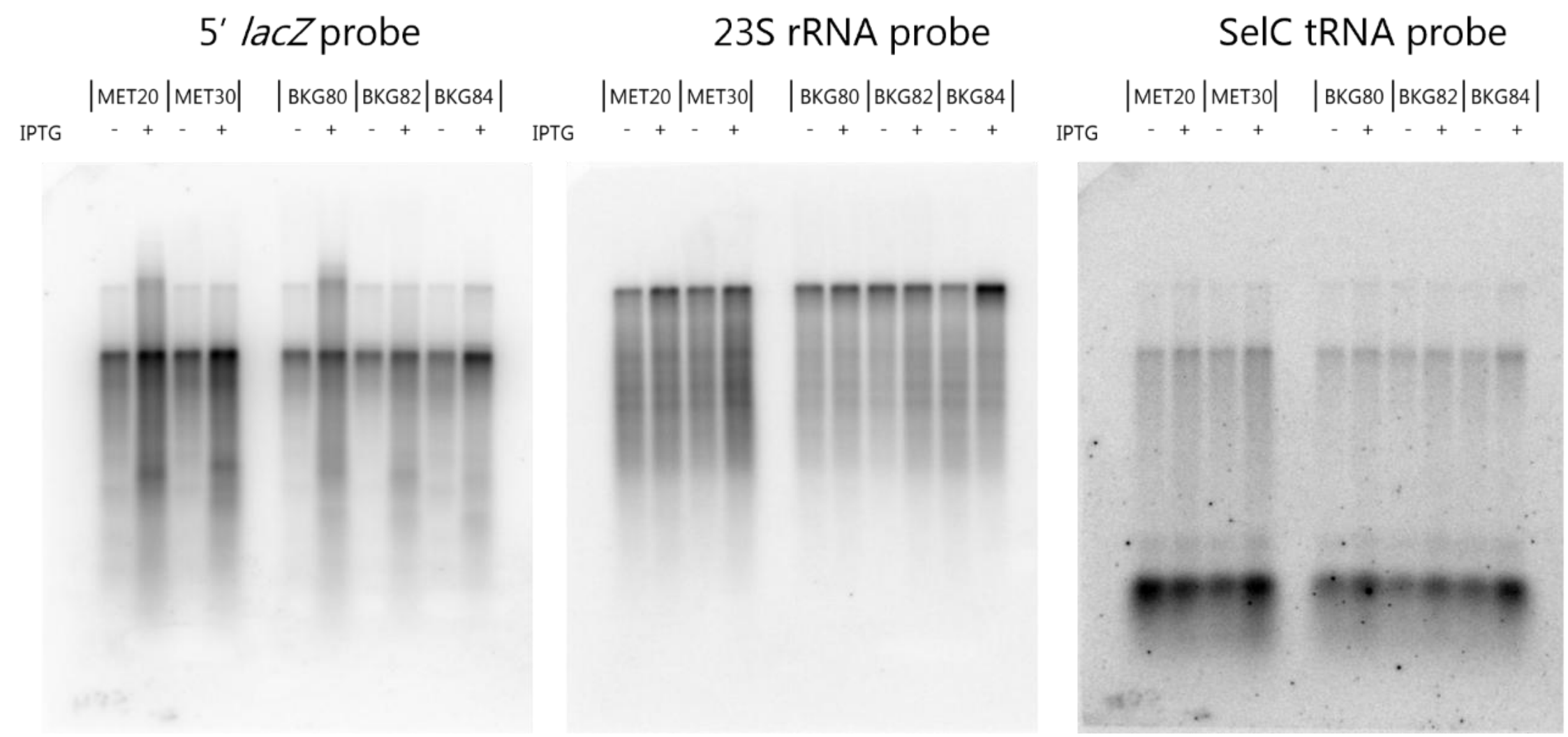

Supplementary Figure S3: Size markers for the northern blot shown in Fig 5b.
The northern blot shown in Fig. 5b of the main text was independently probed for 23S rRNA and $tRNA^{selC}$ to confirm the identity of the ribosomal RNA bands seen with the *5′ lacZ v2* probe and to add the tRNA size marker. $tRNA^{selC}$ is rare in *E. coli* but was abundant in the RNA samples because a whole-cell spike-in culture overexpressing $tRNA^{selC}$ had been added for quantification purposes (5).

Supplementary reference list

1. Pedersen,S., Terkelsen,T.B., Eriksen,M., Hauge,M.K., Lund,C.C., Sneppen,K. and Mitarai,N. (2019) Fast Translation within the First 45 Codons Decreases mRNA Stability and Increases Premature Transcription Termination in E. coli. *J. Mol. Biol.*, **431**, 1088–1097.
2. Thomason,M.K., Bischler,T., Eisenbart,S.K., Förstner,K.U., Zhang,A., Herbig,A., Nieselt,K., Sharma,C.M. and Storz,G. (2015) Global Transcriptional Start Site Mapping Using Differential RNA Sequencing Reveals Novel Antisense RNAs in Escherichia coli. *J. Bacteriol.*, **197**, 18–28.
3. Murakawa,G.J., Kwan,C., Yamashita,J. and Nierlich,D.P. (1991) Transcription and decay of the lac messenger: role of an intergenic terminator. *J. Bacteriol.*, **173**, 28–36.
4. Cannistraro,V.J., Subbarao,M.N. and Kennell,D. (1986) Specific endonucleolytic cleavage sites for decay of Escherichia coli mRNA. *J. Mol. Biol.*, **192**.
5. Prossliner,T., Agrawal,S., Heidemann,D.F., Sørensen,M.A. and Svenningsen,S.L. (2023) tRNAs Are Stable After All: Pitfalls in Quantification of tRNA from Starved Escherichia coli Cultures Exposed by Validation of RNA Purification Methods. *mBio*, **14**, e0280522.
